# Involvement of CD26 in Differentiation and Functions of Th1 and Th17 Subpopulations of T Lymphocytes

**DOI:** 10.1155/2021/6671410

**Published:** 2021-01-20

**Authors:** Xiangli Zhao, Wenhan Wang, Kai Zhang, Jingya Yang, Hendrik Fuchs, Hua Fan

**Affiliations:** ^1^Charité-Universitätsmedizin Berlin, Corporate Member of Freie Universität Berlin, Humboldt-Universität zu Berlin, and Berlin Institute of Health, Institut für Laboratoriumsmedizin, Klinische Chemie und Pathobiochemie, Berlin, Germany; ^2^Institute of Edible Fungi, Shanghai Academy of Agricultural Sciences, Shanghai, China; ^3^College of Food Science and Technology, Shanghai Ocean University, Shanghai, China

## Abstract

CD26, acting as a costimulator of T cell activation, plays an important role in the immune system. However, the role of CD26 in the differentiation of T cell subsets, especially of new paradigms of T cells, such as Th17 and Tregs, is not fully clarified. In the present study, the role of CD26 in T cell differentiation was investigated *in vitro*. CD26 expression was analyzed in the different subsets of human peripheral blood T lymphocytes after solid-phase immobilized specific anti-CD3 mAb stimulation. Here, the percentage of CD4^+^ cells significantly increased and most of these cells were coexpressed with CD26, suggesting a close correlation of CD26 expression with the proliferation of CD4^+^ cells. Subsequently, after immobilized anti-CD3 mAb stimulation, CD26 high-expressing cells (CD26^high^) were separated from CD26 low-expressing cells (CD26^low^) by magnetic cell sorting. We found that the percentages of cells secreting Th1 typical cytokines (IL-2, IFN-*γ*) and Th17 typical cytokines (IL-6, IL-17, and IL-22) or expressing Th17 typical biomarkers (IL-23R, CD161, and CD196) in the CD26^high^ group were markedly higher than in those in the CD26^low^ group. In addition, a coexpression of CD26 with IL-2, IFN-*γ*, IL-17, IL-22, and IL-23R in lymphocytes was demonstrated by fluorescence microscopy. These results provide direct evidence that the high expression of CD26 is accompanied by the differentiation of T lymphocytes into Th1 and Th17, indicating that CD26 plays a crucial role in regulating the immune response.

## 1. Introduction

CD26/DPPIV (dipeptidyl peptidase) is a multifunctional integral type II transmembrane glycoprotein with a broad cell-surface distribution [1]. As serine proteases, DPPIV cleaves the dipeptides after proline or alanine at the penultimate position of the N-terminus of several bioactive peptides and thereby modulates their activities in diverse biological processes [2]. Besides its enzyme activity, CD26 was also shown as a costimulator involved in T cell activation and differentiation by its interaction with other cellular molecules, such as adenosine deaminase (ADA), receptor-type protein tyrosine phosphatase (CD45), CARMA1, and caveolin-1 [3, 4]. The expression of CD26 in T lymphocytes is differentially regulated during T cell development. As an activation marker of T cells, CD26 is mainly expressed on CD4^+^ T cells, and it is thought to be a marker of T helper type 1 cells [4, 5]. Although both Th1 and Th2 cells express CD26, Th1 cells express three- to sixfold more CD26 protein than Th2 cells [6]. Other studies have indicated that CD26 expression induced the cytokine production of Th1 cells, including IL-2, IFN-*γ*, IL-10, and IL-12 [7]. *In vivo*, CD26 deficiency decreased the production of IL-2 and IL-4, delayed the production of IFN-*γ* in sera of mice after pokeweed mitogen (PWM) stimulation, and increased secretion of IL-4, IL-5, and IL-13 in bronchoalveolar lavage (BAL) after ovalbumin-induced airway inflammation [8, 9]. In recent years, a new major effector population of CD4^+^ T cells has been defined and designated as Th17 cells, which play important roles in many diseases [10–12]. One of the Th17 signature cytokines is IL-17 which is a proinflammation factor. Besides IL-17, Th17 cells can produce other proinflammatory cytokines, including IL-22, IL-26, and IFN-*γ*, and recent studies have shown that Th17 cells express IL-23R, lectin-like receptor CD161, and chemokine receptor CCR6 (CD196) [13, 14]. It has been reported that human Th17 cells also express a high level of CD26/DPPIV [15]. However, the role of CD26 in the differentiation of Th17 cells has not been clearly investigated. Besides Th17 cells, regulatory T cells (Tregs) are another subpopulation of T helper cells [16]. Tregs modulate the immune activities through their immunosuppressive effect on other self-reactive T cells thereby contributing to the maintenance of immunologic self-tolerance [17]. Previous studies found that the majority of human Tregs strongly and constitutively express CD25 (CD25^high^), and a fork-head transcription factor (Foxp3) is required for the development and function of CD4^+^CD25^+^ regulatory T cells and regards as one of the specific markers of Tregs [16, 17]. Recently, we have demonstrated a delayed allogeneic skin graft rejection in CD26-deficient mice. During graft rejection, the concentration of IL-17 in serum and the percentage of cells secreting IL-17 in mouse peripheral blood lymphocytes (MPBLs) were both significantly lower while the percentage of regulatory T cells (Tregs) was significantly higher in MPBLs of CD26^–/–^ mice than in those of CD26^+/+^ mice [18]. To further investigate the role of CD26 in the differentiation of Th17 subpopulations of human T lymphocytes, in this work, the correlation of CD26 expression with the differentiation of subsets of human T lymphocytes after solid-phase immobilized specific anti-CD3 mAb stimulation was investigated *in vitro*. We demonstrated that CD26 is closely involved in regulating the differentiation and functions of Th1 and Th17 subpopulations of T lymphocytes.

## 2. Materials and Methods

### 2.1. Separation of Human Peripheral Blood Lymphocytes

Healthy human blood collection was performed according to the German Ethics laws, and approval (EA4/106/13) was obtained from the Ethics Committee of Charité Universitätsmedizin Berlin. Lymphocytes from human peripheral blood were isolated using Ficoll density gradient centrifugation (GE Healthcare, Sweden). The isolation process was performed according to the manufacturer's instructions. Briefly, human peripheral blood was collected and then centrifugated using a simple and rapid centrifugation procedure. Differential migration of cells during centrifugation results in the formation of layers containing different cell types: the bottom layer contains erythrocytes; the layer immediately above the erythrocyte layer contains mostly granulocytes; at the interface between the plasma and the Ficoll-Paque layer, mononuclear cells are found together with other slowly sedimenting particles (e.g., platelets) with low density. We then collect the interface layer of mononuclear cells and culture the cells in the tissue culture-treated dish overnight, and then, the monocytes were adherent to the dish and the lymphocytes were suspended. We then collected the suspension lymphocytes and identify the purity using flow cytometry; more than 95% cells were lymphocytes. The lymphocytes were next cultured in the not tissue culture-treated flasks for further experiment.

### 2.2. Activation of Human Lymphocytes by Stimulation with Solid-Phase Immobilized Anti-CD3-mAb

It has previously been reported that lymphocytes could be activated by stimulation with solid-phase immobilized specific monoclonal anti-CD3 antibodies (mAbs, such as OKT3), in which CD26 was selectively involved in the activation pathway triggered by anti-CD3 [19]. According to Hegen's protocol [19], human peripheral blood lymphocytes (HPBLs) were stimulated by immobilized anti-human CD3 mAb (OKT3, IgG2a) (Thermo Fisher Scientific, USA). Briefly, each of 100 *μ*L PBS with 2 *μ*g/mL anti-CD3 mAb (stimulated group) or without antibodies (PBS, as negative control) was immobilized in a well of 96-well plates overnight. After removal of PBS buffer, 200 *μ*L lymphocyte culture including 2 × 10^5^ fresh isolated lymphocytes in RPMI-1640 growth medium (supplemented with 10% FBS, 100 *μ*g/mL streptomycin, and 100 UI/mL penicillin) was cultured directly in each well of the 96-well plate with or without immobilized antibody at 37°C in a humidified atmosphere with 5% CO_2_ for 72 h.

### 2.3. Measurement of Lymphocyte Proliferation

The proliferation of lymphocytes after stimulation was measured by flow cytometry after cells were labeled with carboxyfluorescein succinimidyl ester (CFSE) assay kit (Thermo Fisher Scientific, USA) according to the instructions of the manufacturer.

### 2.4. Measurement of Cytokine Secretion of HPBLs after Stimulation Using ELISA

Three days after stimulation, the cell culture suspensions of HPBLs were collected. After centrifugation, the supernatant was transferred into new tubes. Different cytokine levels in the supernatant were measured with ELISA kits (R&D Systems, Minnesota, USA). The procedure is according to the instructions of the manufacturer.

### 2.5. Separation of CD26^+^ Cells by Magnetic Cell Sorting (MACS)

MACS MicroBeads (Miltenyi Biotec, Germany) were used for the separation of cells expressing CD26. Lymphocytes were collected at day three after stimulation. At first, the mouse anti-human CD26 mAb (anti-CD26 mAb_350_ prepared in our own laboratory) was used to label the lymphocytes for 1 h at 4°C. Following two washing steps, magnetic MicroBeads labeled with anti-mouse IgG were added to the cells and incubated further for 15 min at 4°C. After a washing step, cells were loaded into the column, which was preplaced in the magnetic field of a suitable MACS Separator (Miltenyi Biotec, Germany). The unlabeled cells were collected after flow-through with two times wash processes. The labeled CD26^+^ cells were bound to the column. After removing the column from the separator and being placed in a suitable collection tube, the labeled CD26^+^ cells were separated from the column and flushed out by help of a plunger. Finally, two groups of cells, the CD26 high-expressing (CD26^high^) group and the CD26 low-expressing (CD26^low^) group, were obtained and then analyzed by flow cytometry.

### 2.6. Analysis of Coexpression of CD26 with Each of the Cytokines or Markers of Different Subpopulations of Lymphocytes by Flow Cytometry

All the cell labeling with immune fluorescence-conjugated antibodies was performed in 1% (*w*/*v*) BSA/PBS at 4°C for 1 h in the dark. For the determination of coexpression of CD26 with cell surface markers of lymphocyte subpopulations, lymphocytes were incubated with both FITC-conjugated anti-human CD26 and PE-conjugated corresponding antibody simultaneously. For determination of the coexpression of CD26 with intracellular cytokines, after incubation with FITC-conjugated anti-human CD26, the cells were washed and fixed with 4% formaldehyde for 5 min, washed again, and subsequently permeabilized with 0.1% Triton X-100 in PBS for 10 min. After further wash steps after permeabilization, the cells were then incubated with PE-conjugated corresponding antibody. Fluorescein isothiocyanate- (FITC-) conjugated and phycoerythrin- (PE-) conjugated antibodies (direct against CD26, CD4, CD8, CD69, CD25, CD71, IL-2, IFN-*γ*, IL-4, IL-13, IL-6, IL-17, IL-22, and IL-23R) as well as allophycocyanin- (APC-) conjugated anti-Foxp3 antibodies were obtained from ImmunoTools (Friesoythe, Germany). Allophycocyanin- (APC-) conjugated anti-CD161 and Per-conjugated anti-CD196 antibodies were provided by MACS Miltenyi Biotec (Bergisch Gladbach, Germany). After the immunofluorescent cells were resuspended in FACS buffer and measured by flow cytometry, the WinMDI 2.9 software was used to analyze the percentages of different lymphocyte subpopulations or cytokine-secreting cells.

### 2.7. Fluorescence Immunomicroscopy

The immunofluorescence staining of cell surface or intracellular proteins was performed as above. Thereafter, cells were resuspended in 20 *μ*L PBS after twice washing steps with PBS and covered on a slide with a thin layer. After air drying, cell layers were added with mounting solution (Thermo Fisher Scientific, USA) and covered by coverslips for fluorescence microscopy. Images were made at a magnification of ×600.

### 2.8. Statistical Analysis

All data represent the mean value ± SD from a minimum of five independent experiments with at least five healthy donor HPBL samples, and each experiment was repeated more than three times. The statistical differences of values were calculated using ANOVA. Differences between groups were considered significant at *p* < 0.05, *p* < 0.01, *p* < 0.005, and *p* < 0.001; *p* values were calculated with a chi-square test.

## 3. Results

### 3.1. Part of the Lymphocytes Was Activated and Proliferated, and the Expression of CD26 Was Upregulated after Antigen Stimulation

**Figure 1:**
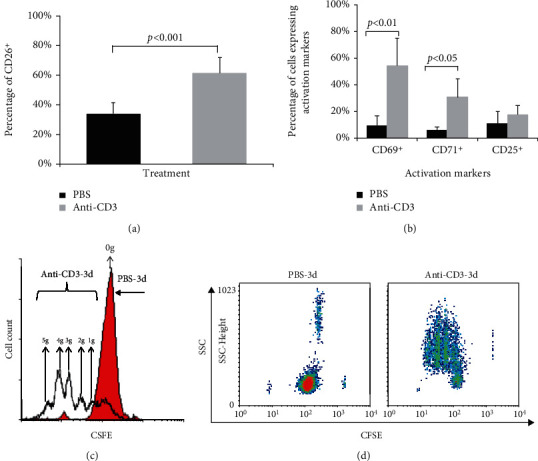
The activation and proliferation of T lymphocyte after stimulation. After 72 h of the stimulation with immobilized anti-CD3 mAb, the activation of lymphocytes was determined by the measurement of the expression of T lymphocyte activation markers (CD69, CD71, CD25, and CD26). The proliferation of lymphocytes was analyzed by CFSE assay. PBS-treated cells were used as controls. (a) Percentages of CD26^+^-HPBLs in the control group and the stimulated group. (b) Percentages of CD69^+^-, CD71^+^-, and CD25^+^-HPBLs in the control group and the stimulated group. Data represented mean value ± SD from a minimum of 5 independent experiments with at least 5 healthy donor HPBL samples, and each experiment was repeated more than 3 times. *p* values were calculated with a chi-square test. (c) Histogram of the proliferated generations of lymphocytes after stimulation by immobilized anti-CD3 mAb (anti-CD3) or only PBS as control (PBS) for three days. The shaded histogram represents the original generation (0g) of the PBS control group at day 3. The hollow histogram indicates the increased 5 generations (1g, 2g, 3g, 4g, and 5g) of the stimulated group three days after stimulation. (d) The dot plots show the proliferated generations of lymphocytes analyzed by flow cytometry.

After the isolation of mononuclear cells and monocyte removal, 24 h, 48 h, and 72 h after stimulation by solid-phase immobilized specific anti-CD3 mAb (OKT3, Thermo Fisher Scientific, USA), the expression level of CD26 was tested, and we found that the CD26 expression level was the highest at day 3 after stimulation (Supplementary Figure 1). Three days after stimulation, the survivability of the cells was tested using Annexin V/PI; we can see that more than 98% cells were alive (Supplementary Figure 2), which can be used for the next study. Then, the activation of HPBLs was determined by the measurement of expression of different lymphocyte activation markers (CD69, CD25, CD71, and CD26). In comparison to nonactivated control cells, the percentage of CD26^+^ HPBLs was significantly increased after stimulation by 85% (33 ± 8%*vs*. 61 ± 14% of total HPBLs, *p* < 0.001) (Figure 1(a)), while the percentages of CD69^+^ and CD71^+^ cells were 6-fold and 5-fold compared to control cells (54.29 ± 20.87%*vs*. 9.07 ± 7.28%, *p* < 0.01; 30.6 ± 14%*vs*. 5.8 ± 2.46%, *p* < 0.05), respectively, and the percentage of CD25^+^ HPBLs was 68% higher than the value in the control group (17.65 ± 6.58%*vs*. 10.49 ± 9.41%) (Figure 1(b)). These results indicate that a substantial part of the HPBLs was activated after stimulation with immobilized anti-CD3 mAb.

To determine the proliferated new generations of lymphocytes after stimulation, the CFSE assay was used. As shown in Figures 1(c) and 1(d), at day three after stimulation, the stimulated group (hollow black histogram) showed five additional peaks that represent five increased generations of HPBLs whereas the PBS control group (shaded red histogram) showed only one peak remaining in the original position, indicating that no new generation was generated. These results provide evidence that the lymphocytes proliferated and increased by up to five new generations after stimulation compared to the lymphocytes of the PBS control group that had not proliferated within three days.

### 3.2. Increased Percentages of CD4^+^-, CD4^+^CD26^+^-, and CD8^+^CD26^+^-HPBLs after Stimulation

**Figure 2:**
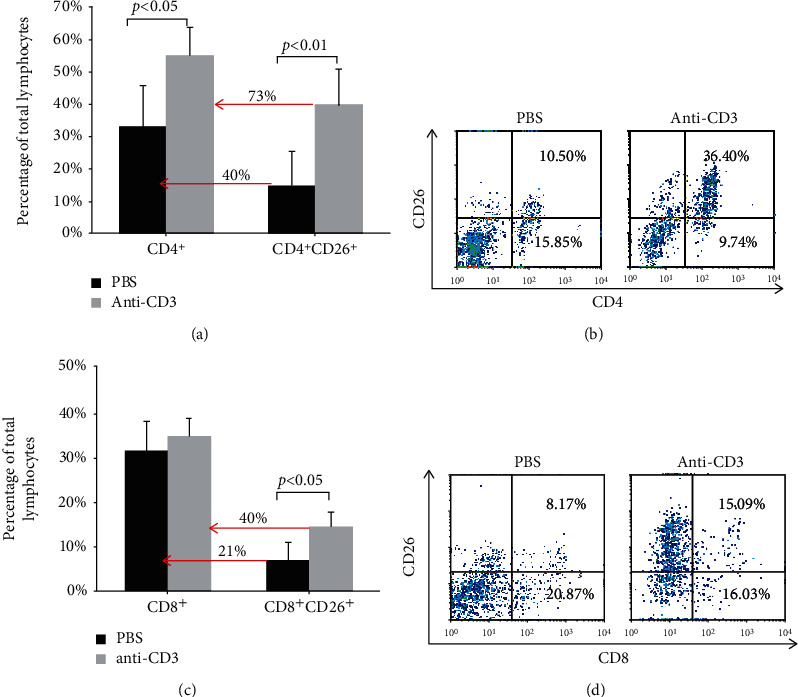
Percentages of CD4^+^ and CD8^+^ cells and cells coexpressing each of these surface markers with CD26 after stimulation. (a) Percentages of CD4^+^ and CD4^+^CD26^+^ cells in the control group and the stimulated group. (c) Percentages of CD8^+^ and CD8^+^CD26^+^ cells in the control group and the stimulated group. Data represented mean value ± SD from five independent experiments with five healthy donor HPBL samples, and each experiment was repeated more than three times. *p* values were calculated with a chi-square test. The dot plots show one typical experiment for the analysis of (b) percentages of CD4^+^ and CD4^+^CD26^+^ cells and (d) percentages of CD8^+^ and CD8^+^CD26^+^ cells by flow cytometry.

In order to clarify the role of CD26 in lymphocyte differentiation, the percentages of CD4^+^ T lymphocytes (T helper cells) and CD8^+^ T lymphocytes (T cytotoxic cells) as well as the percentage of cells that were coexpressing each of these two subpopulation markers with CD26 were analyzed after stimulation. As shown in Figure 2, after stimulation, the percentage of CD4^+^ cells was increased from 32.57 ± 8.91% to 54.72 ± 12.85% of total HPBLs while the percentage of CD8^+^ cells did not increase significantly. This result suggests a strong proliferation of the T helper subpopulation (CD4^+^) of T lymphocytes after stimulation. Further analysis revealed that after stimulation the percentage of cells that were coexpressing CD4 and CD26 (CD4^+^CD26^+^) in total HBPLs was 2.8-fold of that in the control group (39.98% *vs*. 14.43%). In the stimulated CD4^+^ subpopulation, about 73% of the CD4^+^ cells were coexpressed with CD26, while in the control CD4^+^ subpopulation only 40% of the CD4^+^ cells were coexpressed with CD26 (Figures 2(a) and 2(b)). As previously known, CD26 is a costimulator of T cell activation; the increased T helper cells (CD4^+^) after stimulation were mostly coexpressed with CD26 observed in the present work, indicating that the activation and proliferation of CD4^+^ cells are closely related to CD26 expression.

While the percentage of CD8^+^ cells did not increase significantly after stimulation, we found that the percentage of CD8^+^CD26^+^ cells in the stimulated group was about 2.1 times than that of the control group (14.28 ± 3.35%*vs*. 6.72 ± 4.21%). In the stimulated group, approx. 40% of CD8^+^ cells were coexpressing CD26, compared with 21% of the CD8 cells in the control group (Figures 2(c) and 2(d)). The increased CD8^+^CD26^+^ cells suggest that CD26 is also related to the activation of CD8^+^ cells. Interestingly, the percentage of total CD8^+^ cells was not increased significantly. Since cell survival analysis showed that almost no dead lymphocytes were observed after stimulation (data not shown), it suggests that T cytotoxic CD8^+^ cells hardly proliferated, or their proliferation rate was much slower than that of CD4^+^ cells.

### 3.3. Higher Percentages of CD4^+^, CD4^+^CD26^+^, and CD8^+^CD26^+^ Cells in the CD26^high^ Group

**Figure 3:**
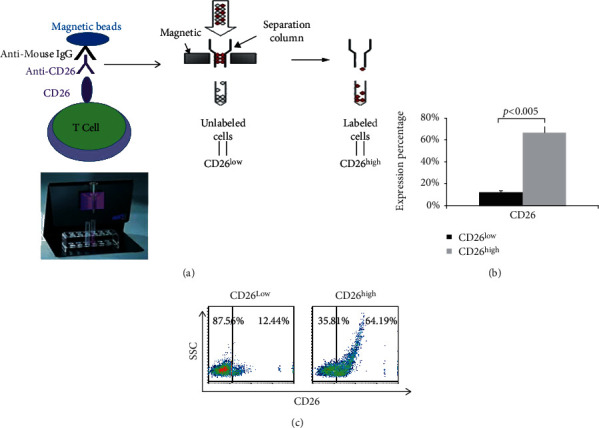
Separation of CD26^low^ and CD26^high^ lymphocytes by magnetic cell sorting (MACS). (a) Procedure of separation of CD26^low^ and CD26^high^ lymphocytes by MACS. After stimulation for three days, lymphocytes were labeled with the mouse anti-human CD26 mAb (anti-CD26 mAb_350_ prepared in our own laboratory) for 1 h at 4°C. Following the two washing steps, magnetic MicroBeads labeled with anti-mouse IgG were added to the cells and incubated further for 15 min at 4°C. After a washing step, cells were loaded into the column which was preplaced in the magnetic field of a suitable MACS Separator (Miltenyi Biotec, Germany). The unlabeled cells were collected after flow-through with two times wash processes. The labeled CD26^+^ cells were bound to the column and then flushed out after removing the column from the separator by help of a plunger. (b) Analysis of CD26 expression in the CD26 high-expressing (CD26^high^) group and the CD26 low-expressing (CD26^low^) group by flow cytometry. Data represented mean value ± SD from a minimum of five independent experiments with at least five healthy donor HPBL samples. (c) The dot plots show one typical experiment for analysis of CD26 expression.

**Figure 4:**
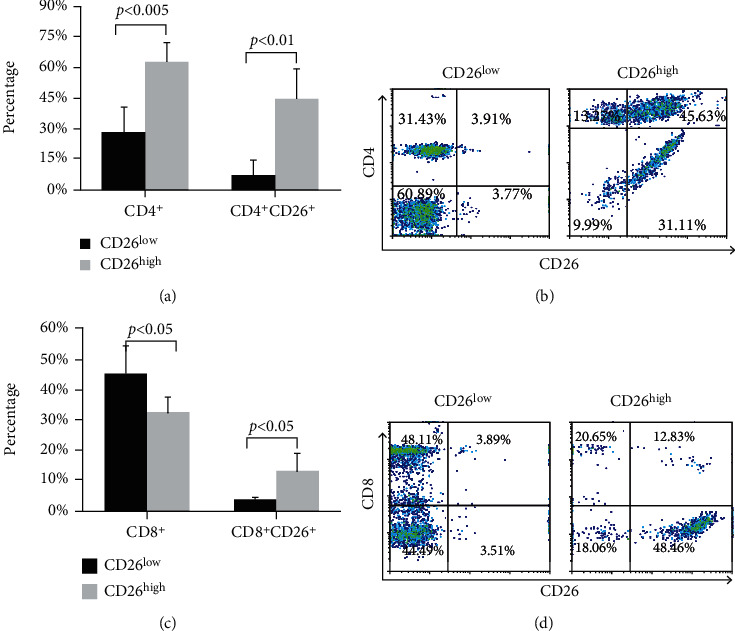
Percentages of CD4^+^, CD8^+^, CD4^+^CD26^+^, and CD8^+^CD26^+^ cells in the CD26^low^ and CD26^high^ groups. (a) Percentages of CD4^+^ and CD4^+^CD26^+^ cells in the CD26^low^ and CD26^high^ groups. (c) Percentages of CD8^+^ and CD8^+^CD26^+^ cells in the CD26^low^ and CD26^high^ groups. Data represented mean value ± SD from seven independent experiments with seven healthy donor HPBL samples, and each experiment was repeated more than three times. Dot plots show the percentages of (b) CD4^+^ and CD4^+^CD26^+^ cells and (d) CD8^+^ and CD8^+^CD26^+^ cells in the CD26^low^ and CD26^high^ groups.

For further analysis of the correlation of CD26 to T cell differentiation, after stimulation, CD26^+^ cells were separated using MACS MicroBeads conjugated with anti-mouse IgG after binding of CD26^+^ lymphocytes with anti-human CD26 mAb (Figure 3). After separation, two groups of cells were obtained: CD26 low-expressing group (CD26^low^) and CD26 high-expressing group (CD26^high^). The expression profiles of CD4^+^ and CD8^+^ and their coexpression with CD26 on surfaces of cells in the CD26^low^ and CD26^high^ groups were analyzed. As shown in Figure 4, the percentage of CD4^+^ cells in the CD26^high^ group was 2.2-fold of that in the CD26^low^ group (62.70 ± 14%*vs*. 28.28 ± 9%, *p* < 0.005), while the percentage of CD8^+^ cells was lower in the CD26^high^ group compared to the CD26^low^ group (32.24% ± 5%*vs*. 45.11 ± 9%, *p* < 0.05). Further analysis showed that the percentage of CD4^+^CD26^+^ cells in the CD26^high^ group was 6-fold of that in the CD26^low^ group (44.27 ± 15%*vs*. 7.13 ± 7%, *p* < 0.01) (Figures 4(a) and 4(b)), while the percentage of CD8^+^CD26^+^ cells in the CD26^high^ group was about 3.5-fold of that in the CD26^low^ group (12.93 ± 6%*vs*. 3.72 ± 0.9%, *p* < 0.05) (Figures 4(c) and 4(d)). These results showed that the expression of CD26 occurred mostly in T helper cells (CD4^+^) and only a small part of T cytotoxic cells (CD8^+^) expressed CD26 after stimulation, indicating activation of most T helper cells (CD4^+^) but only a few T cytotoxic cells (CD8^+^). In consideration of the greatly increased percentages of CD4^+^ cells and CD4^+^CD26^+^ cells after stimulation, CD26 is closely involved in the proliferation of T helper cells (CD4^+^) undoubtedly.

### 3.4. Higher Secretion of Th1 and Th17 Typical Cytokines or Expression of Th17 Molecular Markers in Cells of the CD26^high^ Group

**Figure 5:**
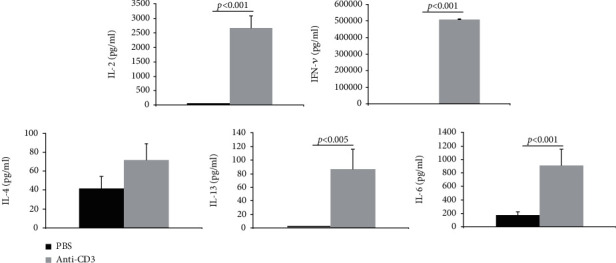
The cytokine secretion profiles of HPBLs after stimulation. Three days after stimulation by immobilized anti-CD3 mAb, the cell culture suspensions of HPBLs were collected. After centrifugation, the supernatant was transferred into new tubes. Different cytokine levels in the supernatant were measured with ELISA kits. The values represent the mean value ± SD of samples from a minimum of 7 healthy donors in each group.

After three days of stimulation, the levels of different cytokines were measured by ELISA. From Figure 5, we can see that after stimulation, great amounts of IL-2, IFN-*γ*, and IL-6 were produced.

The level of IL-13 also increased after stimulation, but it was very limited, while the secretion level of IL-4 did not increase significantly. As known, IL-2 and IFN-*γ* are mainly secreted by Th1 cells, and IL-4 and IL-13 are mainly secreted by Th2 cells. Although IL-6 is mainly separated by macrophage during acute inflammation, more and more reports suggested that IL-6 is also secreted by T cells, such as Th17.

**Figure 6:**
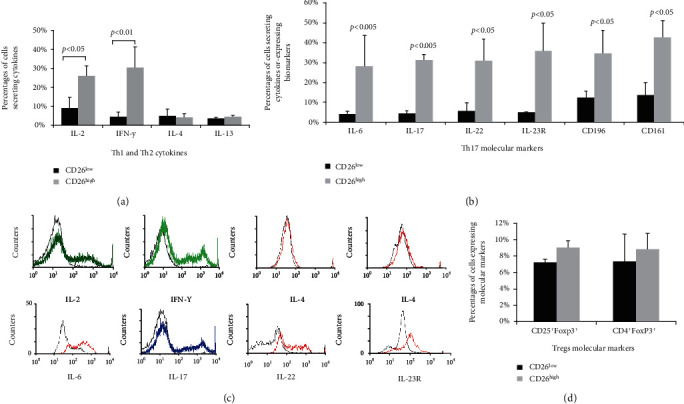
Percentage of cells secreting different cytokines in the CD26^low^ and CD26^high^ groups. After separation, the cells in the CD26^low^ group and the CD26^high^ group were labeled with different monoclonal antibodies against cytokines or surface markers at 4°C for 30 min and then measured by flow cytometry. (a) The percentages of cells secreting Th1 and Th2 typical cytokines in the CD26^low^ and CD26^high^ groups. (b) The percentages of cells secreting Th17 typical cytokines or expressing Th17 typical biomarkers in the CD26^low^ and CD26^high^ groups. (c) Overlay histograms demonstrate the relative expression of cells secreting different cytokines in the CD26^low^ and CD26^high^ groups. The black line indicates the values of CD26^low^ group cells while the color lines indicated the values of CD26^high^ group cells. (d) The percentages of cells expressing Tregs typical biomarkers in the CD26^low^ and CD26^high^ groups. Data represented mean value ± SD from a minimum of five independent experiments with at least five healthy donor HPBL samples, and each experiment was repeated more than three times.

To investigate the association of CD26 expression with CD4 cell differentiation, the percentages of T helper subpopulations were determined by flow cytometry after cells were labeled with fluorescence-conjugated antibodies against corresponding cytokines or cell surface markers. The results showed that the percentages of cells secreting Th1 typical cytokine IL-2 and IFN-*γ* in the CD26^high^ group were significantly higher than those in the CD26^low^ group (Figure 6(a)). The percentage of cells secreting IL-2 in the CD26^high^ group was approximately three times than that of the CD26^low^ group (25.93 ± 5.39%*vs*. 8.89 ± 5.85%), and the percentage of cells secreting IFN-*γ* in the CD26^high^ group was about seven times than that of the CD26^low^ group (30.17 ± 11.14%*vs*. 4.45 ± 2.63%). Similarly, in Figure 6(b), the percentages of cells secreting Th17 typical cytokines (IL-6, IL-17, and IL-22) or expressing biomarkers (IL-23R, CD196, and CD161) were evidently higher in the CD26^high^ group than in the CD26^low^ group. The percentages of cells secreting IL-6 or lL-17 in the CD26^high^ group were about 7-fold of those in the CD26^low^ group (28.11% *vs*. 4.12%, 31.28% *vs*. 4.32%). The percentage of cells secreting IL-22 in the CD26^high^ group was 5.4-fold compared to that in the CD26^low^ group (31.05% *vs*. 5.74%). The percentage of cells expressing IL-23R was even higher in the CD26^high^ group, 7-fold of that in the CD26^low^ group (35.93% *vs*. 4.98%). In addition, the percentages of cells expressing Th17 surface biomarkers CD196 and CD161 in the CD26^high^ group were 2.8-fold and 3-fold of those in the CD26^low^ group (34.73% *vs*. 12.35%, 42.52% *vs*. 13.59%), respectively. Histogram analysis showed that the expression levels of Th1 and Th17 typical cytokines (IL-2, IFN-*γ*, IL-6, IL-17, and IL-22) or a Th17 typical surface marker (IL-23R) in the cells of the CD26^high^ group were much higher in relation to the cells of the CD26^low^ group (Figure 6(c)). These results suggest that the expression of CD26 is involved in the regulation of the differentiation and functions of Th1 and Th17 subpopulations of T lymphocytes.

On the other hand, the percentages of cells secreting Th2 typical cytokines either IL-4 or IL-13 showed exceptionally low and no significant differences between the CD26^high^ group and the CD26^low^ group (Figure 6(a)). Similarly, the histogram analysis showed that there were no significant differences in the expression levels of Th2 cytokines (IL-4 and IL-13) in cells between the CD26^high^ group and the CD26^low^ group (Figure 6(c)). In addition, the percentages of cells expressing molecular markers of regulatory T cells (CD25^+^Foxp3^+^ or CD4^+^Foxp3^+^) in the CD26^high^ group did not have significant differences to those in the CD26^low^ group (Figure 6(d)). These results suggest that the CD26 expression is not correlated to the differentiation and functions of Th2 and Treg subpopulations of T lymphocytes after antigen stimulation.

### 3.5. Coexpression of CD26 with Th1 or Th17 Typical Cytokines in Cells of the CD26^high^ Group

**Figure 7:**
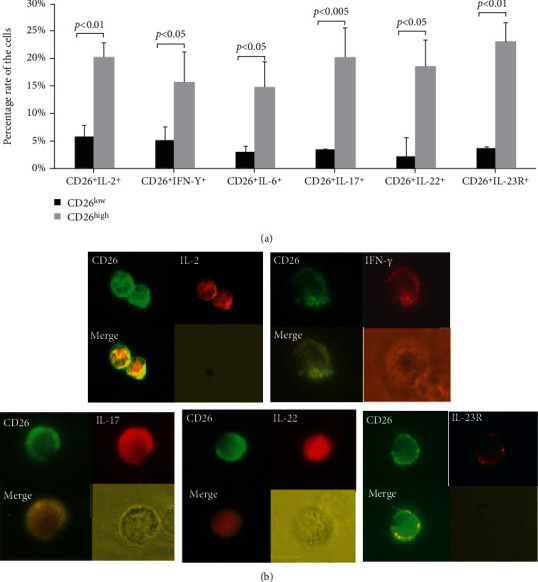
Coexpression of CD26 with each of the Th1 or Th17 typical cytokines or surface markers in the cells of the CD26^low^ and CD26^high^ groups. Lymphocytes were harvested at 72 h after stimulation and were double-stained with the FITC-conjugated anti-CD26 mAb and PE-conjugated anti-IL-2, anti-IFN-*γ*, anti-IL-17, anti-IL-6, anti-IL-22, or anti-IL-23R mAb. (a) Percentages of cells coexpressing CD26 with each of the Th1 typical cytokines (IL-2 or IFN-*γ*), Th17 typical cytokines (IL-6, IL-17, and IL-22), or Th17 typical surface marker (IL-23R) in the CD26^low^ and CD26^high^ groups. Data represented mean value ± SD from a minimum of five independent experiments with at least five healthy donor HPBL samples, and each experiment was repeated more than three times. (b) Coexpression of CD26 with Th1 or Th17 typical biomarkers was observed by fluorescence microscopy. Images were made at ×600 magnifications. Coexpression of CD26 with IL-2, IFN-*γ*, IL-17, IL-22, or IL-23R in some lymphocytes indicated by the merged images.

The association of CD26 expression to the differentiation of Th1 or Th17 subset was further analyzed by determination of the coexpression of CD26 with each of the Th1 typical cytokines (IL-2 or IFN-*γ*), Th17 typical cytokines (IL-6, IL-17, and IL-22), or Th17 specific surface marker (IL-23R). In comparison to the CD26^low^ group, the percentages of cells that were coexpressing CD26 with each of these cytokines were obviously higher in the CD26^high^ group (Figure 7(a)). The percentages of cells that were coexpressing CD26 with IL-2 (CD26^+^IL-2^+^) or IFN-*γ* (CD26^+^IFN-*γ*^+^) in the CD26^high^ group were 3.5- and 3-fold of those in the CD26^low^ group (20.31% *vs*. 5.83% and 15.66% *vs*. 5.18%), respectively. Notably, the percentages of cells that were coexpressing CD26 with IL-17 (CD26^+^IL-17^+^), IL-6 (CD26^+^IL-6^+^), or IL-22 (CD26^+^IL-22^+^) in the CD26^high^ group were nearly 6-fold, 5-fold, and 6.5-fold of those in the CD26^low^ group (20.14% *vs*. 3.43%, 14.81% *vs*. 3%, and 18.64% *vs*. 2.86%), respectively. Also, the percentage of cells that were coexpressing CD26 with Th17 marker IL-23R (CD26^+^IL-23R^+^) in the CD26^high^ group was 6-fold compared to that in the CD26^low^ group (23.14% *vs*. 3.7%) (Figure 7(a)).

Fluorescence microscopy detected that the CD26 protein was predominantly located on the cell plasma membrane, while IL-2, IFN-*γ*, IL-17, and IL-22 were present in the cytosol and IL-23R was also mainly located on the cell surface. After merging the photos, CD26 was found to be coexpressed with IL-2, IFN-*γ*, IL-17, IL-22, or IL-23R (Figure 7(b)) in the same lymphocytes. Since IL-2 and IFN-*γ* are typical Th1 cytokines, the coexpression of Th1 cytokines with CD26 suggests a correlation of CD26 to the differentiation and function of Th1 cells. Similarly, IL-17 and IL-22 are typical Th17 cytokines, and IL-23R is a typical Th17 cell surface marker. Therefore, the coexpression of Th17 cytokines or markers with CD26 suggests a correlation of CD26 to the differentiation and function of Th17 cells.

## 4. Discussion

CD26 was determined as one of the costimulators for T cell activation [3, 4], and the costimulatory effect of CD26 for T cell activation could be mediated by the interaction of CD26 with the ecto-adenosine deaminase (ADA), tyrosine phosphatase CD45, CARMA1, or caveolin-1 [20, 21]. In the present work, antigens of lymphocytes were stimulated by using an immobilized anti-CD3 mAb (OKT3, IgG2a) to further investigate the role of CD26 in T cell differentiation. Three days after stimulation, the activation of lymphocytes was determined by the enhanced expression of lymphocyte activation markers CD26, CD69, CD71, and CD25 (Figures 1(a) and 1(b)). CD69 is one of the earliest cell surface antigens expressed by T cells following activation. It acts as a costimulatory molecular and surface marker for T cell activation and proliferation. CD71 (the transferrin receptor) and CD25 (the IL-2 receptor alpha chain) are the other two molecular surface markers of T cell activation and proliferation [22, 23]. The significant increase in the expression of CD69, CD71, and CD25 indicates that most of the lymphocytes are activated after stimulation [23]. In addition, CD26 expression was also significantly upregulated after stimulation (Figure 1(a)) suggesting an association of CD26 to the activation of T lymphocytes, which is consistent with previous studies [3, 4]. After stimulation, the coexpression level of CD26 with CD4^+^ or CD8^+^ was increased markedly (Figure 2), which indicates that the expression of CD26 is related not only to the activation of CD4^+^ cells but also to a certain extent to the activation of CD8 cells. A previous study reported that a unique pattern of CD26 high expression was identified on influenza-specific CD8^+^ T cells but not on CD8^+^ T cells specific for cytomegalovirus, Epstein Barr virus, or HIV, which suggested that high CD26 expression may be a characteristic of long-term memory cells [24]. A later study indicated that CD26^+^CD8^+^ cells belong to the early effector memory T cell subsets. The CD26-mediated costimulation of CD8^+^ cells provokes effector function via granzyme B, tumor necrosis factor-*α*, IFN-*γ*, and Fas ligand [25]. The role of CD26 in the differentiation and function of CD8^+^ cells needs further investigation.

Thereafter, the proliferation of lymphocytes was analyzed after stimulation. It was found that in comparison to lymphocytes without stimulation (PBS control), which did not proliferate, the immobilized anti-CD3 mAb stimulated lymphocytes proliferated up to five generations (Figures 1(c) and 1(d)). Further analysis showed that after stimulation, the percentage of CD4^+^ cells in total HPBLs was increased significantly while the percentages of CD8^+^ did not change (Figure 2). The upregulated percentage of CD4^+^ cells suggests that the immobilized anti-CD3 mAb triggered mainly the proliferation of CD4^+^ lymphocytes [26]. It was found that the percentage of CD4^+^ cells in the CD26^high^ group was significantly higher than that in the CD26^low^ group, and most of the CD4 cells were coexpressed with CD26 (Figures 4(a) and 4(b)). Previously, Ohnuma et al. have reported that CD26 was thought to be mostly expressed by memory T helper cells, and its expression was preferential on CD4^+^ cells and associated with T cell activation as a costimulatory molecule [4]. Blockade of CD26-mediated T cell costimulation with soluble caveolin-1 induced anergy in CD4^+^ cells [20]. Besides studies on the involvement of CD26 in the activation and proliferation of CD4^+^ T cells *in vitro*, *in vivo* investigation using CD26 knockout mice presented a decreased percentage of CD4^+^ cells [8]. CD4^+^ cells are T helper cells and they can secrete different cytokines upon T cell activation, and these cytokines play a crucial role in the activation and/or proliferation of other effector cells, such as B cells, cytotoxic T cells, and macrophages [27, 28]. The higher percentage of CD4^+^ cells in the CD26^high^ group and CD26 high expression in activated CD4^+^ cells observed in the present work further confirm that CD26 expression is involved not only in the activation but also in the proliferation and in further bioprocesses and functions of CD4^+^ cells.

After activation, CD4^+^ cells proliferate and differentiate into different subpopulations. Th1 and Th2 are the two main and earliest defined subpopulations of T helper cells [27]. Th1 cells can potentially produce large amounts of IFN-*γ* and IL-2 cytokines while Th2 effector cells are characterized by the production of IL-4 and IL-13 [28]. In the current work, after three days of stimulation with immobilized anti-CD3 mAb, a large amount of IL-2, IFN-*γ*, and IL-6 was detected in cell culture by ELISA analysis, while the levels of IL-13 and IL-4 were very low (Figure 5). After cell sorting of CD26-expressing cells, the percentages of cells secreting each of Th1 typical cytokines IFN-*γ* and IL-2 in the CD26^high^ group were significantly higher than those in the CD26^low^ group (Figures 6(a) and 6(c)). Moreover, most of the cells secreting IFN-*γ* or IL-2 were coexpressing CD26 (Figure 7). In a previous study, the upregulation of CD26 expression on CD4^+^ cell surfaces was identified to be related to the production of Th1 cytokines [4]. It was reported that the solid-phase immobilized anti-CD26 mAb had a comitogenic effect by inducing CD4^+^ lymphocyte proliferation and enhancing IL-2 production in conjunction with submitogenic doses of anti-CD3 [19]. The inhibitor of DPPIV/CD26 enzyme activity has been suggested to be able to reduce the production of IL-2, IL-6, and IFN-*γ* of human and mouse T cells under mitogen stimulation [7]. Supporting these findings, the results of the present work showed that the expression of CD26 is associated with the differentiation of Th1 cells. Th1 is an important subset of T helper cells. The positive relation between the activation of CD4^+^ cells and CD26 expression (Figures 4(a) and 4(b)) benefits the differentiation of CD4^+^ cells into a Th1 subset.

Interestingly, the percentages of cells secreting Th2 typical cytokines IL-4 or IL-13 were not only very low (<5%) in the CD26^low^ and CD26^high^ groups, but they also did not present any difference between both kinds of cell groups (Figure 6(a)). As one of the main subpopulations of T helper cells, the Th2 subset is often recognized as an opposite of Th1 cells since Th2 cytokines may suppress the activity and proliferation of Th1 cells during immune responses [29]. Our results indicate that CD26 expression is not related to the differentiation of CD4^+^ cells into the Th2 subset after antigen stimulation.

Besides Th1 and Th2 subsets, Th17 and Tregs are the other two important subsets of T helper subpopulations. Th17 is a more recently identified subset of CD4^+^ cells [10], which is distinct from classic Th1 and Th2 subsets [11, 30]. These cells originate from naive CD4^+^ precursor cells mainly in the presence of TGF-*β* and IL-6, and their differentiation requires IL-23 [13, 14]. As a novel member of the CD4^+^ T subset, it is important to clarify the role of CD26 in the differentiation and function of Th17 cells. After cell sorting, the percentage of cells secreting Th17 typical cytokines (IL-17 and IL-22) or expressing Th17 molecular markers (IL-23R, CD161, and CD196) was found to be significantly higher in the CD26^high^ group than in the CD26^low^ group (Figure 6(b)). Moreover, most of the cells secreting IL-17 and IL-22 or expressing IL-23R, CD161, and CD196 were coexpressed with CD26 (Figure 7). This indicates an involvement of CD26 in the differentiation of CD4^+^ cells into the Th17 subset. A previous study showed that Th17 cells express a high level of CD26, and the phenotypic analysis of Th17 cells could be identified by the CD26 expression [15]. Th17 cells play an important role in preventing the pathogen invasion through secreting proinflammatory cytokines. Clinical research found that CD26 was related to some diseases which involved the immune response initiated by Th17 cells through inducing chronic inflammation or autoimmunity, like rheumatoid arthritis and multiple sclerosis [31].

Recently, it has been reported that inhibition of the enzyme activity of CD26 by sitagliptin reduced the proliferation and Th1/Th17 differentiation of human lymphocytes *in vitro* [32], and the CD26 costimulatory blockade improves lung allograft rejection and is associated with enhanced IL-10 expression *in vivo* [33]. We have also shown recently that CD26 deficiency resulted in a delayed allogeneic skin graft rejection after allogeneic skin transplantation. The concentrations of serum IgG, including its subclasses IgG1 and IgG2a, were significantly reduced in CD26^–/–^ mice during graft rejection. The secretion levels of the cytokines IFN-*γ*, IL-2, IL-6, IL-4, and IL-13 were significantly reduced whereas the level of the cytokine IL-10 was increased in the serum of CD26^–/–^ mice compared to CD26^+/+^ mice. Additionally, the concentration of IL-17 in serum and the percentage of cells secreting IL-17 in mouse peripheral blood lymphocytes (MPBLs) were both significantly lower while the percentage of regulatory T cells (Tregs) was significantly higher in MPBLs of CD26^–/–^ mice than in those of CD26^+/+^ mice [18]. In line with the results of these *in vivo* experiments, the results of the present *in vitro* study confirm that the expression of CD26 is not only highly correlated to the differentiation of Th1 and Th17 but also plays an important role in the functions of Th1 and Th17. It is precisely because CD26 plays an indispensable role in the differentiation and function of Th1 and Th17 lymphocytes, which results in a lack of effective Th1 and Th17 cells when CD26 is absent under relevant pathological conditions. The present study provides more insight into the role of CD26 for the function of Th17 cells and related diseases and will support future research in this field.

It is reported that CD26 can be used as a negative selection marker for Tregs [34]. In the present study, the percentages of Tregs were very low in the CD26^high^ and CD26^low^ groups, and no significant difference was found between the two groups (Figure 6(d)), indicating that the expression of CD26 is not necessary for the differentiation of Tregs after immobilized anti-CD3 mAb stimulation.

In conclusion, CD26 is not only an activation marker for T lymphocytes, but its expression is closely related to the subsequent proliferation, differentiation, and functions of T lymphocytes. Considering that the balance between Th1 and Th2 and the balance between Th17 and Tregs play a prominent role in immune responses [35, 36], our results in this study demonstrated that the high expression of CD26 is beneficial to the differentiation of T lymphocytes into Th1 and Th17 subpopulations after antigen stimulation, indicating a crucial role of CD26 in regulating the immune response to inflammation and autoimmune reactions. The correlation of CD26 with the differentiation balance between Th1 and Th2 and between Th17 and Tregs observed in this study provides more insights into the role of CD26 in related diseases. The important role of CD26 in immune regulation suggests that it would become a therapeutic target for related diseases [37].

## Data Availability

This manuscript describes original work, and neither the entire nor any part of its content has been published previously or has been accepted elsewhere. The presented version (https://www.authorea.com/users/364553/articles/484935-involvement-of-cd26-in-differentiation-and-functions-of-th1-and-1-th17-subpopulations-of-t-lymphocytes) is just preprint and never accepted or published.

## Abbreviations

HPBL: Human peripheral blood lymphocyte
mAb: Monoclonal antibody
DPPIV: Dipeptidyl peptidase IV (CD26)
Tregs: Regulatory T cells.

## Supplementary Materials

Supplementary Materials Supplementary Figure 1: analysis of the CD26 expression level at different time points (24 h, 48 h, and 72 h) after immobilized anti-CD3 mAb stimulation. After the isolation of mononuclear cells by Ficoll, monocytes were removed by cell adhesion. Lymphocytes were collected from suspensions and stimulated with immobilized anti-CD3 mAb for 24 h, 48 h, or 72 h. The CD26 expression level was measured at indicated time points by flow cytometry. Supplementary Figure 2: analysis of the survival rate of lymphocytes after immobilized anti-CD3 mAb stimulation using FITC-Annexin V/PI Assay. (A) The lymphocytes were collected at 72 h after immobilized anti-CD3 mAb stimulation or PBS treatment. The cell survival rate was analyzed by flow cytometry after FITC-Annexin V/PI staining. Data are shown as mean value ± SD of five separated experiments. (B) The data shown is a typical representative of five experiments.
