# Supplementary material for: Involvement of CD26 in Differentiation and Functions of Th1 and Th17 Subpopulations of T Lymphocytes
Source: J Immunol Res. 2021 Jan 20;2021:6671410. doi: 10.1155/2021/6671410 (PMC7843192; doi:10.1155/2021/6671410)
Supplement: Supplementary Materials — Supplementary Figure 1: analysis of the CD26 expression level at different time points (24 h, 48 h, and 72 h) after immobilized anti-CD3 mAb stimulation. After the isolation of mononuclear cells by Ficoll, monocytes were removed by cell adhesion. Lymphocytes were collected from suspensions and stimulated with immobilized anti-CD3 mAb for 24 h, 48 h, or 72 h. The CD26 expression level was measured at indicated time points by flow cytometry. Supplementary Figure 2: analysis of the survival rate of lymphocytes after immobilized anti-CD3 mAb stimulation using FITC-Annexin V/PI Assay. (A) The lymphocytes were collected at 72 h after immobilized anti-CD3 mAb stimulation or PBS treatment. The cell survival rate was analyzed by flow cytometry after FITC-Annexin V/PI staining. Data are shown as mean value ± SD of five separated experiments. (B) The data shown is a typical representative of five experiments. [file 6671410.f1.docx]

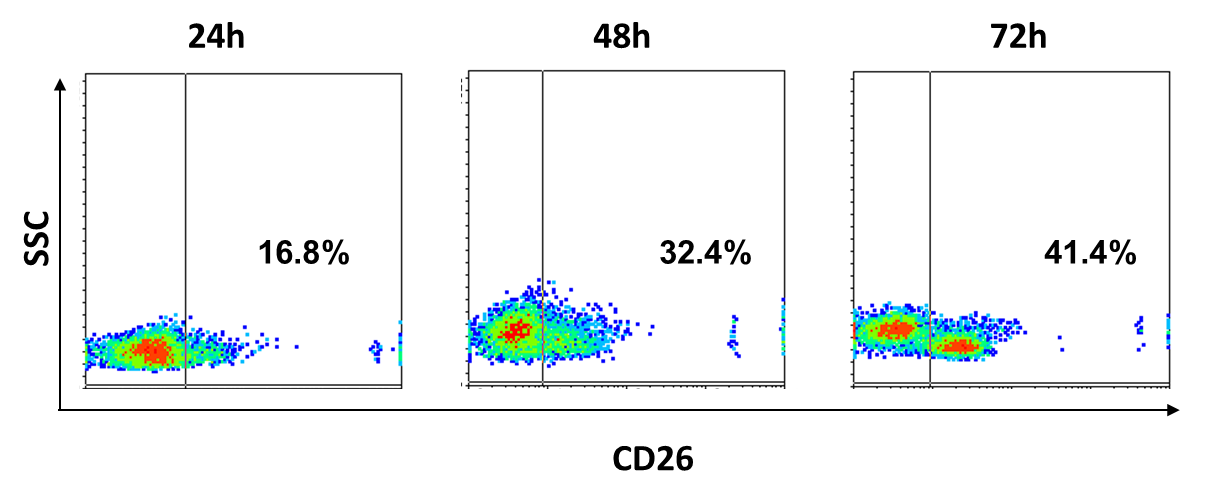


**Supplementary Figure 1: Analysis of the CD26 expression level at different time points (24h, 48h, and 72h) after immobilized anti-CD3 mAb stimulation.**

After the isolation of mononuclear cells by Ficoll, monocytes were removed by cell adhesion. Lymphocytes were collected from suspensions and stimulated with immobilized anti-CD3 mAb for 24h, 48h, or 72h. The CD26 expression level was measured at indicated time points by flow cytometry.


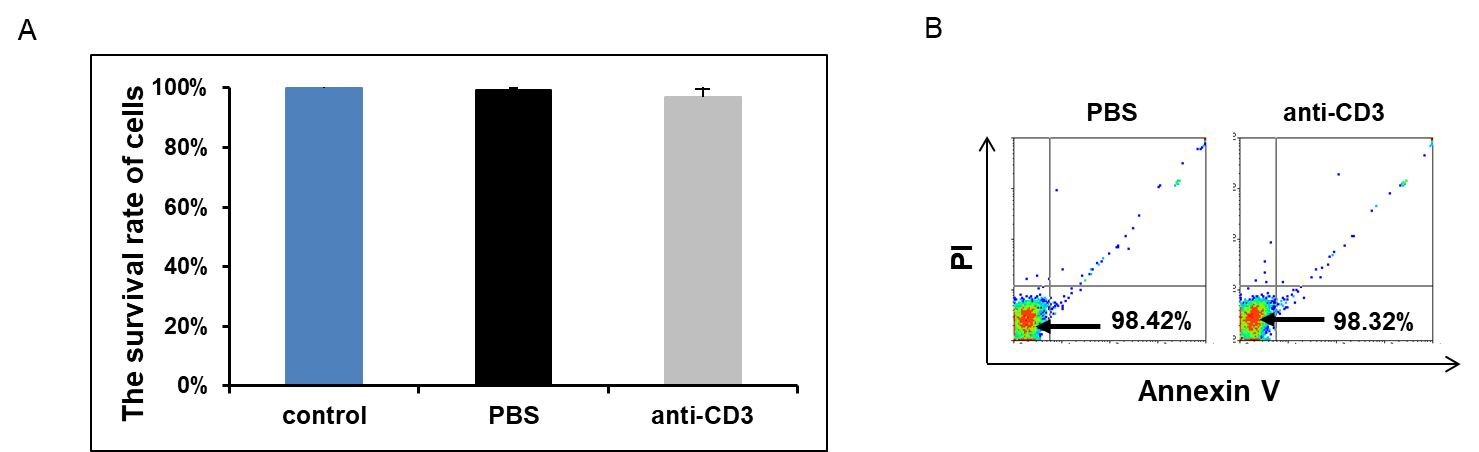


**Supplementary Figure 2: Analysis of the survival rate of lymphocytes after immobilized anti-CD3 mAb stimulation using FITC-Annexin V/PI Assay.**

(A)The lymphocytes were collected at 72 h after immobilized anti-CD3 mAb stimulation or PBS treatment. The cell survival rate was analyzed by flow cytometry after FITC-Annexin V/PI staining. Data are shown as mean value ± SD of five separated experiments.

(B)The data shown is a typical representative of five experiments.
